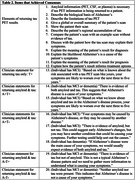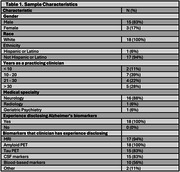# Expert consensus on communicating tau PET results: Findings from a modified Delphi study

**DOI:** 10.1002/alz70861_108446

**Published:** 2025-12-23

**Authors:** Claire M Erickson, Kyra O’Brien, Emily A. Largent, Kristin Harkins, Cameron Coykendall, Melanie Kleid, Justin Clapp, Jason Karlawish

**Affiliations:** ^1^ Banner Alzheimer's Institute, Phoenix, AZ USA; ^2^ University of Pennsylvania Perelman School of Medicine, Philadelphia, PA USA; ^3^ University of Pennsylvania, Philadelphia, PA USA; ^4^ Penn Memory Center, Perelman School of Medicine at the University of Pennsylvania, Philadelphia, PA USA

## Abstract

**Background:**

As the clinical utility of tau PET increases, it will be integrated into care for cognitively impaired persons. Unfortunately, clinicians presently lack guidance for communicating tau PET results.

**Method:**

We conducted a modified Delphi process with US‐based clinicians with expertise in human tau PET imaging, identified via strategic literature search. A modified Delphi process is a means of obtaining reliable expert consensus when empirical evidence does not exist. Individual interviews with clinician‐experts elicited their views on when and how to return tau PET results. From this, we derived candidate approaches and statements for return of tau PET results. Delphi participants rated their agreement with these items in two rounds of online surveys to achieve consensus on acceptable approaches to communicating tau PET results. Consensus was defined as ≥80% of respondents rating the acceptability of a statement on a five‐point Likert as “4‐somewhat” or “5‐highly.”

**Result:**

Twenty clinician‐experts were interviewed; of these, eighteen completed both survey rounds (Table 1). Ultimately, clinician‐experts reached consensus on 12 elements of returning tau PET results – such as showing the PET scan and describing its limitations – as well as eight statements for relaying results to patients: one when returning tau alone, three for concordant amyloid and tau results (i.e., A+T+, A‐T‐) and four for discordant amyloid and tau results (i.e., A+T‐, A‐T+) (Table 2). Experts agreed amyloid information (PET, CSF, or plasma) is necessary if tau PET information is being returned to a patient. Through comments, clinician‐experts highlighted the importance of communicating regional accumulation when interpreting tau PET results, emphasizing its utility over communicating measures of global accumulation. Notably, experts preferred the ternary terminology “elevated/intermediate/not elevated” or “elevated in neocortex/elevated in MTL/not elevated” (*n* =14) over binary terminology (“elevated/not elevated”) (*n* =4) when providing a global interpretation of tau PET.

**Conclusion:**

Tau PET is poised for clinical adoption due to the unique diagnostic and prognostic information it provides, yet, guidance for returning tau PET results is currently lacking. The results from our modified Delphi process address a critical gap in the literature and provide novel guidance.